# A Quest for an Eco-centric Approach to International Law: the COVID-19 Pandemic as Game Changer

**DOI:** 10.1007/s42439-020-00031-0

**Published:** 2020-11-11

**Authors:** Sara De Vido

**Affiliations:** 1 Ca’ Foscari University of Venice, Venice, Italy; 2 Manchester International Law Centre, Manchester, UK

**Keywords:** Anthropocentrism, Ecocentrism, Animal rights, Rights of the nature, Environment, Global health, COVID-19, Paradigm change, Ecofeminism

## Abstract

This Reflection starts from the ongoing COVID-19 pandemic as unprecedented occasio to reflect on the approach to international law, which—it is contended—is anthropocentric, and its inadequacy to respond to current challenges. In the first part, the Reflection argues that there is, more than ever, an undeferrable need for a change of approach to international law toward ecocentrism, which puts the environment at the center and conceives the environment as us, including humans, non-human beings, and natural objects. To encourage the incorporation of ecocentrism in the entire discipline, the Reflection will rely on some insight of ecofeminism, whose potential has not been fully investigated in international legal scholarship. In the second part, the Reflection illustrates what an eco-centric international law would mean, imagining three possible applications: first, what the author has called environmental global health, which is connected to the current pandemic and puts into question the proposals dealing with global health that completely miss the theorization of the environment as a whole; second, how actors of international law would change according to an eco-centric perspective; and, third, how the rules prohibiting the use of force might be reconceptualized. The analysis contained in these pages cannot itself exhaust all the possible nuances of the legal reasoning, but it is aimed at being a provocative starting point for a change in the mindset and approach of international legal scholarship.

## Introduction: Challenging the Anthropocentric Structure of International Law

Back in 1826, Mary Shelley imagined a virus that would have annihilated the world in the last decade of the twenty-first century in *The Last Man*, the least known of her works. It was a novel, but one with a high power of evocation. In 1994, the American science author Laurie Garrett wrote *The Coming Plague*, in which she scientifically talked about deforestation and disruption of natural balances, viruses whose existence becomes notorious with the outbreak of epidemics, and social disparities in the access to health services. What can international lawyers learn from these Cassandran books?

The pandemic that spread around the world in 2020 has affected human rights and raised human security issues. It represents, above all, a major environmental issue. International law, as all fields of law, is facing enormous challenges. How relevant is international environmental law in the debate? Are states, along with international organizations, capable of providing appropriate answers? This Reflection does not aim to suggest possible ways of surviving (legally speaking) this global pandemic or to determine the responsibility for the spread of the coronavirus. It rather starts from the pandemic as unprecedented *occasio*^1^ to reflect on the approach to international law, which—it is contended—is anthropocentric, and its inadequacy to respond to current challenges. In the first part, the Reflection argues that there is, more than ever, an undeferrable need for a change of approach to international law toward ecocentrism, which puts the environment at the center and conceives the environment as “us,” including humans, non-human beings, and natural objects. If eco-centric moves have been encouraged in environmental law, scholarship has not taken a step further to include eco-centric considerations in all fields of international law. To encourage the incorporation of ecocentrism in the entire discipline, the Reflection will rely on some insight of ecofeminism, whose potential has not been fully investigated in international legal scholarship. The aim is not to illustrate or to critically discuss ecofeminist theories but to use some of their pivotal arguments to unravel the anthropocentric nature of international law.

In a second part, the Reflection illustrates what an eco-centric international law would mean, imagining three possible applications: first, what I have called “environmental global health,”^2^ which is connected to the current pandemic and puts into question the proposals dealing with global health that completely miss the theorization of the environment as a whole; second, how actors of international law would change according to an eco-centric perspective; and, third, how the rules prohibiting the use of force might be reconceptualized. The analysis contained in these pages cannot itself exhaust all the possible nuances of the legal reasoning, but it is aimed at being a provocative starting point for a change in the mindset and approach of international legal scholarship. It is an argument in process to provoke a debate in legal scholarship and identify possible areas of future research.

## Ecocentrism and International Environmental Law

Ecocentrism is not new to international law. Over the past decades, there have been some eco-centric moves in international environmental law, which disrupted its initial anthropocentric biases based on the idea of protecting the environment “not for its own sake, but because of its value to humans – its importance for human health, economics, recreation, and so forth.”^3^ Starting from the World Charter on Nature of 1982, the UN GA has adopted a series of Harmony with Nature resolutions, stressing the coexistence of humankind in harmony with nature.^4^ A quite peculiar treaty, also in terms of the subjects that negotiated and signed it, is represented by the 2017 Whanganui Agreement in New Zealand, which has included the perspective of the river as a holistic system.^5^ This eco-centric move contributes to disrupting patterns of oppression within human beings and in the relationship of humanity with nature because it grants participation to the indigenous communities and is guided by the rights—and not by the human property interests that lay upon—of this natural object. Moving to the regional level, the Inter-American Court of Human Rights, in a landmark advisory opinion of 2017 and the contentious case decided in February 2020, derived an autonomous right to a healthy environment from Article 26 of the American Convention of Human Rights.^6^ In the court’s view, the human right to a healthy environment protects nature, even absent evidence of possible risks for human beings, because of its importance for the rest of living beings.^7^ This constitutes an unprecedented move in regional jurisprudence: appreciating how the right to a healthy environment clearly serves the humans, in an anthropocentric way, but it also goes beyond that to conceive the environment as the balanced relationship among natural objects and the humans.^8^ Another eco-centric development is represented by the advent of the rights of nature, incorporated in the so-called environmental constitutionalism, represented by countries such as Ecuador which inserted a chapter on *Pachamama* as part of the 2008 constitutional revision.^9^ These developments should not be underestimated, because they have emphasized the centrality of nature in the legal reasoning. They challenged laws that have been created to protect the environment for the benefit of human beings without considering two key aspects: first, that human beings are themselves part of the nature, and second, that the existence of nature per se, independently from human beings, matters. However, nature is neglected in other fields of international law.

What if we consider international law in its entirety as anthropocentric and blind to the dynamics of powers and domination that are present in the international community, within human communities and between the humans and the environment? Patterns of discrimination and oppression characterized by both intra-species and inter-species hierarchies are entrenched in international law and can be appreciated by developing some ecofeminist concerns and arguments.

## Patterns of Domination and Oppression in International Law: an Ecofeminist Perspective

International feminist legal scholarship has already pointed out the patriarchal structure of international law as we know it today, characterized by the marginalization of women in the international legal system. As Chinkin and Charlesworth wrote in *The Boundaries of International Law*, the exclusion of women is “an integral part of the structure of the international legal order, a critical element of its stability,” and the silences of the discipline are “as important as its positive rules and rhetorical structures.”^10^ International legal discourse has been informed by dichotomies, including the culture/nature one.^11^ Dianne Otto, using a queer feminist analysis, has stressed the limits of human rights law, which has not questioned the understanding of sex/gender as dualistic (m/f),^12^ and “the gendered, raced, imperial, heteronormative, privileged, autonomous and ableist assumptions implicit in this ‘universal’ subject – the human who is able to fully enjoy human rights and fundamental freedoms.”^13^

Patterns of oppression and domination are however not only intra-species but also inter-species, in the relation between humans and the nature. The human/nature dichotomy has been used to determine patterns of oppression and discrimination beyond humans, including non-human animals and “natural objects.”^14^ The law, as the earth Jurisprudence has pointed out,^15^ has been theorized in a specific association human/nature, where the former dominates the latter.^16^ Ecological disasters and pandemics are the tip of the iceberg of a long process of destruction. This process has been called “slow violence,” meaning “a violence that occurs gradually and out of sight, a violence of delayed destruction that is dispersed across time and space, an attritional violence that is typically not viewed as violence at all.”^17^ What we experience now has been the product of decades of environmental exploitation and domination of human beings, the “privileged” one,^18^ on the natural environment. The concepts we use in international law inevitably incorporate “traces of power and domination,”^19^ and this acknowledgement allows us to reflect on possible non-dominant changes. As argued by the Special Rapporteur on the right to a healthy environment, David Boyd, “today’s dominant culture and the legal system that supports it are self-destructive. We need a new approach rooted in ecology and ethics […] We are part of nature: not independent, but interdependent.”^20^

Ecofeminism has played a pivotal role in denouncing the patterns of oppression within humans and of a part of humanity on nature, despite presenting different streams of thought. As acknowledged by the philosopher Plumwood, “ecological feminists differ on how and even whether women are connected to nature, on whether such connection is in principle sharable by men, on how to treat the exclusion of women from culture, and on how the revaluing of the connection with nature.”^21^ Even though the description of ecofeminism is beyond the scope of this article, it is worth recollecting that the word was coined by Françoise d’Eaubonne in a work of 1974, “Le féminisme ou la mort,” where she highlighted the environmental costs of development and argued that overpopulation of the planet was caused by the patriarchal refusal of women’s self-determination on their bodies.^22^ Sheila Collins contended, in the same year, that patriarchy rested on “racism, sexism, class exploitation, and ecological destruction”^23^ and soon after Rosemary Radford Ruether encouraged the union between the demands of the women’s movement and the ecological one.^24^ Seeds of ecofeminism can be found well before 1970s in Rachel Carson, who was a pioneer in unravelling the disconnections between human beings and the environment and paved the way for the work of ecofeminism worldwide.^25^ Ecofeminism has also confronted the issue of non-human animals’ suffering and incorporated it into a larger critique of the ill treatment of the natural world.^26^

Ecofeminism does not simply mean to combine environmental and feminist issues. As Puleo argued, it is “an attempt to outline a new utopian horizon, addressing the environmental issue from the categories of patriarchy, androcentrism, care, sexism and gender.”^27^ She also stressed how environmentalism is not always feminist and how, in turn, feminism does not necessarily demonstrate “great ecological sensitivity.”^28^ The dialogue between feminism and environmentalism is pivotal to stress the impact of environmental degradation on gender and the contribution of women to put at the center of any (including legal) reasoning the nature.^29^ The founding trait of ecofeminism, namely, oppression and domination, is particularly interesting for the scope of this Reflection to denounce, on one hand, the weaknesses of the international legal system, and, on the other hand, to propose valuable alternatives. Why has ecofeminism been overlooked from an international legal point of view? Malone illustrated the very few contributions in international law dealing with ecofeminism and found a possible reason of this attitude in the specialization of international law into sub-issues, which miss the complete picture of the analysis.^30^ At the same time, however, to play the devil’s advocate, it should also be acknowledged that neither ecofeminism nor environmental humanities have seriously taken into account the legal discipline.^31^ Resting on the insight of ecofeminism, the Reflection will now propose an eco-centric change of perspective which should embrace all fields of international law.

## What an Eco-centric International Law Would Look like

The eco-centric approach to the international law that is encouraged in these pages could be informed by some of the main findings of ecofeminism, most importantly the need for a disruption of patterns of oppression and of the dichotomy human/nature or culture/nature, and the emphasis placed on the intersections of grounds of oppression.^32^ Issues such as climate change and global health—as the pandemic has been ruthlessly telling us—should consider these intersections and should put the environment at the very center of any legal discussion. Two authors talked about “locating nature” in international law, going beyond the understanding of nature as serving limited interests and as a matter of international environmental law only.^33^ They argued that the natural environment is not incidental to international law and that nature is “a fundamental driver of disciplinary evolution, shaping legal concepts in seminal ways.”^34^ This Reflection uses the concept of “environment” rather than of “nature,” which includes natural objects and human and non-human beings, where humans are not the dominant factor but rather a part of a holistic whole. The idea of the environment as “us” was introduced by Christopher Stone in his pioneering article and book almost fifty years ago: “because the health and well-being of [human]kind depend upon the health of the environment, these goals will often be so mutually supportive that one can avoid deciding whether our rationale is to advance ‘us’ or a new ‘us’ that includes the environment.”^35^ Ecocentrism is considered the encompassing idea that disrupts the human/nature divide and considers the relationships between organisms and the healthy interaction of all components of ecosystems, including human beings. The adjective “healthy” stresses that ecocentrism does not mean a complete absence of interference with nature or that humanity cannot defend itself from lethal viruses.^36^ It considers the inherent value of nature and recognizes the holistic and mutual relationship of humanity with it.^37^ It also purports to disrupt, in line with ecofeminist theories, the patterns of domination and oppression which lead to the exploitation of nature by a part of the humanity. Ecocentrism is not the mere opposite of anthropocentrism, with the risk of proposing a new dichotomy in place of the disrupted one; it rather seeks to reconceive the relationship among the different elements of the environment. This relationship would lead to a refreshing, and probably provocative, look at the most traditional concepts of international law.

## An Eco-centric Approach to International Law in Practice

A change of approach in international law cannot be thoroughly explained in few pages. This paragraph will sketch some possible trajectories which will constitute a solid starting point for future research. It starts from what I have called “environmental global health,” before moving to two selected, and admittedly limited, issues of international law, namely, actors of international law and the prohibition of the use of force.

### Environmental Global Health

The current framework of global health law, mainly based on World Health Organization (WHO) law, seems inadequate to respond to current challenges, because it does not appreciate the importance of taking into consideration the environment as “us.”^38^ The environment has always been at the margin of legal reasoning in health issues, either not considered (as in the WHO International Health Regulations) or diminished to the role of determinant of the right to health. The alternative proposed in these pages is the concept of “environmental global health,”^39^ defined as the system of actors, including non-human animals and natural objects, and legal instruments, measures, and policies, aimed at the prevention, protection, and response to transboundary health and environmental issues, taking into consideration social and economic disparities, and going beyond the humans to address imbalances of natural ecosystems.^40^ An eco-centric approach appreciates the connections between global health, the human right to health, and the environment.^41^ More than a human right to a healthy environment, what is needed is the recognition of every human, non-human being, and ecosystem’s right to be part of a healthy environment, where prevention is fundamental, and where all actions are taken in light of their impact on the environment.

An environmental global health governance should focus on prevention, ensuring campaigns to raise awareness, for example, of how wet markets represent ticking bombs for the environment, but also of how increasing emissions, plastics, disruption of ecosystems, land grabbing, water grabbing, weak public health systems characterized by inequalities and lack of participation, fuel the spread of pandemics and/or the effects of them. As the reality has ruthlessly shown us, the prevention of pandemics cannot be merely based on the WHO law. What we need is the sharing of responsibilities and liabilities for a plurality of actors^42^: not only states but also international organizations, transnational corporations, and even individuals. It is time to consider that the prevention and response to pandemics cannot be realized by states, as traditionally conceived in international law, only.^43^ In pragmatic terms, this would mean, for example, that the achievement of the cut of emissions of CO2 is a treaty obligation to counter climate change and an obligation that states and non-state actors must abide by to guarantee the individual human right to health and environmental global health. One might contend that the notion of environmental global health is itself anthropocentric. However, even though more rigorous understandings of ecocentrism might sound ideal, they do not grasp the existing patterns of oppression within human society, and the role a part of humanity has played in the destruction of ecosystems. Ecofeminism has been able to capture the essence of oppression, which should lead to the reconceptualization of well-known categories of international law.^44^

### Actors of International Law

States represent anthropocentrism in their reproduction of rooted schemes of subordination. Scholarship has emphasized the male nature of the state as traditionally conceived and identified the elements of oppression, including the principle of territorial integrity.^45^ Judge Cançado Trindade argued that the end of “the monopoly of international personality by states and the expansion of such personality at international level is a guarantee against the abuses of the past, reducing at international level the scope for oppression or tyranny.”^46^ International institutions have marginalized women, minorities, indigenous, and LGBT groups. In an eco-centric perspective, inspired by ecofeminist arguments, states are no longer the main subjects of international law. The approach this Reflection proposes decenters states to include several actors that contribute to the existence of the environment as “us.” The aforementioned Whanganui Agreement of 2017, for example, demonstrates that eco-centric considerations are not impossible to grasp from a legal point of view and that they should be pursued.^47^ Cases have been brought in front of national courts on behalf of rivers and other elements of nature,^48^ and the Inter-American Court of Human Rights has elaborated a concept of right to a healthy environment which is eco-centric because it looks at the equilibrium of the nature irrespective of the effects of its degradation on human beings.^49^ A further step forward was undertaken in a transitional justice setting. The Colombia’s peace jurisdiction, Jurisdicción Especial para la Paz, has recently decided that the Katsa Su and Cxhab Wala Kile territories, belonging to the indigenous Awá and Nasa peoples, have been victims of the Colombia civil war.^50^ The jurisdiction has the mandate to investigate some relevant cases of the conflict that lasted 50 years. Among the seven cases chosen by the jurisdiction, case 2 concerned the complaints put forward by Awá people, Afro-descendent communities, and mestizo rural communities, who stressed “that [their territory] has identity and dignity that constitute it as a subject of rights.”^51^ The Colombian jurisdiction has specifically acknowledged the connection between the people and the territory, by contending in its resolution that “for some indigenous peoples, the experiences of the war are not defined only by the damage caused to people, as the consequences are written as well into the vision of beings that live […] in the same natural environment.”^52^ This decision leads to a reconsideration not only of actors of international law but also of reparations and the rules on (states’) responsibility. To what extent is a state responsible for the damages to the environment, when the latter is conceived as composed not only of human beings, but also of non-humans and natural objects? How will reparations be defined as a consequence of the affirmation of state responsibility? The preservation or restoration of biodiversity, for example, can be considered a form of reparations, irrespective of whether human interests have been affected. Habitat restoration projects can also support the protection of non-human animals, on whose rights the debate has been quite controversial.^53^ It is worth acknowledging that in the 1970s, Peter Singer, in his famous *Animal Liberation*, argued that discrimination of living beings “solely on account of their species is a form of prejudice, immoral and indefensible in the same way that discrimination on the basis of race is immoral and indefensible.”^54^ Singer compared specism to racism, and feminists identified the connections between specism and sexism. In that respect, the radical feminist Catherine MacKinnon denounced how “women have been animalized, animals feminized, often at the same time.”^55^ To disrupt the binary category human (or better, some humans)/nature, with the dominance of the former on the latter, a legal revolution is needed. Boyd argued that “the progress in scientific understanding and the concomitant evolution of societal values seem to compel movement in this direction,” as demonstrated by the legal recognition that “animals deserve significantly stronger rights than they have been granted in the past.”^56^ The real nature of animals is one of complex individuals, “living in elaborate social networks, relationships, and communities,”^57^ and the law needs to catch up with the science, spurring societal change. Anne Peters elaborated a concept of global animal law to promote animal rights, invoking five main reasons, the main one being “the advantage of having rights as opposed to being merely beneficiaries of standards of conduct […]. The recognition of rights carries with it a powerful message of prima facie inviolability.”^58^ In other words, “animal rights would […] preclude the current routine sacrifice of fundamental animal interests in favor of trite human interests.”^59^

Decentering states and the humans does not simply mean to re-center the legal debate on nature, most importantly because states and (a part of) human beings have been the cause of the ongoing environmental disasters and ecological imbalances. An eco-centric approach would rather entail that human policies and perspectives are reoriented toward a common end, with the guarantee of the integrity of the environment, to which humans constitute an integral, though no longer dominant, part. It would also stress the importance of non-governmental organizations and environmental (and not only human) rights defenders in the disruption of the predominance of states in the international realm.

### The Prohibition of the Use of Force

An eco-centric reading of the prohibition of the use of force can follow two (at least initial) directions.^60^ The first one rests on the marginalization of the environment in the provisions of UN Security Council resolutions. Hence, for example, in a recent resolution on peacekeeping,^61^ the UN body only recognized that “possible adverse effects of environmental deterioration may, in the long run, aggravate certain existing threats to the stability of some host states,” but did not consider that the action of peacekeepers must respect the environment, considered as a broad concept encompassing human and non-human animals, along with natural objects, as contended in this article. Another example is the UN Security Council Resolution No. 2379 of 2017, establishing the investigative team to support domestic efforts to hold ISIL accountable by collecting, preserving, and storing evidence in Iraq of acts that may amount to international crimes committed by the ISIL, which does not mention the environment at all. The Security Council condemned “the commission of acts by ISIL (Da’esh) involving murder, kidnapping, hostage-taking, suicide bombings, enslavement, sale into or otherwise forced marriage, trafficking in persons, rape, sexual slavery and other forms of sexual violence, recruitment and use of children, attacks on critical infrastructure, as well as its destruction of cultural heritage, including archaeological sites, and trafficking of cultural property,” but not the impact of the group’s actions on the environment.^62^

It is also the main argument of this paragraph that when authorizing the use of force, the UN Security Council should include the respect for the environment, especially due to air and soil contamination caused by military strikes that affects the ecosystems and the human beings as part of them. It would be even possible to say, evoking the call for disarmament and peace of the International Congress of Women of 1915, that if we consider the environment as a whole, the quest for peace manifests itself as expression of ecocentrism.

The second direction concerns the use of weapons. In this sector, the eco-centric approach to international law can be appreciated at its best, suggesting an eco-centric, inspired by ecofeminism, rewriting of the highly criticized Advisory Opinion on the legality of the threat or use of nuclear weapons of 1996.^63^ It is well-known that in a split 7-7 decision, with the President casting the final vote, the court on one hand recognized that the threat or use of nuclear weapons “would generally be contrary to the rules of international law applicable in armed conflict, and in particular the principles and rules of humanitarian law” and, on the other hand, could not conclude definitively “whether the threat or use of nuclear weapons would be lawful or unlawful in an extreme circumstance of self-defense.” The environment was mentioned by the court in a key paragraph, in which it pointed out that “the environment is under daily threat and that the use of nuclear weapons could constitute a catastrophe for the environment” and that “the environment […] the living space, the quality of life and the very health of human beings.” The environment is however more than the living space of human beings; it is the place of all the elements conceived as a whole. A partial rewriting of the opinion in an eco-centric perspective will consider the applicable rules on the environment as combined with international human rights law, arguing that the use of nuclear weapons—potentially all weapons—have an impact on the right to a healthy environment, which is no longer a simple “human” right, but it is the right of all the species and ecosystems that live in it. Arguing that the use or threat of the use of nuclear weapons had to be judged illegal under all circumstances, Judge Weeramantry in his dissenting opinion came close to an eco-centric view, pointing out that these weapons not only contradicts human dignity but also “endangers the human environment in a manner which threatens the entirety of life on the planet.”^64^

## Concluding Remarks

We did not need the pandemic to highlight the weaknesses of the global health governance and international law more generally as we conceive them today. New ways of framing international law have been advocated for a long time. The pandemic represents however the game changer, which unveils existing problems and pushes for a change. The interconnection between global health and the environment must be appreciated in all ideas of reform of the WHO system as a starting point, but this does not seem enough. There is an urgent need of new creative approaches to international law to grasp the complexity of the environment, which includes human beings as part of a holistic whole. This argument does not only lead to the adoption of new rules of international law but also to apply and interpret existing ones in an eco-centric way. This Reflection has provided a few examples of existing practice in the described direction. Its invitation to endorse an eco-centric approach to international law, which is critical of existing patterns of discrimination and oppression, is not devoid of risks, including the resistance states could show to new rules informed by eco-centric concerns. Nonetheless, as international lawyers, we should respond to the crisis and demonstrate that international law, starting from its basic concepts, can evolve and embrace the environment, which is, in the end, nothing less than a pivotal issue to work on in all the fields of the discipline.

## Footnotes

1 *Occasio* should be meant in these pages as “opportunity.” It means that the pandemic, despite the negative consequences in the entire world, must be seen as a “ground for action or feeling.” From Old French (thirteenth century), it is meant as “cause, reason, excuse, pretext” and from Late Latin “cause” (from the online etymology dictionary https://www.etymonline.com/).

2 See in this sense, De Vido (forthcoming), and a complete list of references on the topic.

3 See, in that sense, Gillespie (Gillispie 1997; Gillispie 2014); Taylor (1998); and Bodansky et al. (2008). See also Emmenegger and Tschentscher (1994).

4 See the most recent resolution on the topic: GA Res 74/224 (Jan 30, 2020).

5 Boyd (2017), 139.

6 Inter-American Court of Human Rights: OC-23/17 Advisory Opinion (Nov 15, 2017), and Aboriginal Community of *Lhaka Honhat v Argentina,* Series C No. 400, *Merits, reparations and costs (Feb 6, 2020)*.

7 OC-23/17 Advisory Opinion, para. 180.

8 See Boyle (2007) and Francioni (2010).

9 On this topic, from a constitutional and comparative law perspective, see, among others, Hayward (2000), Gellers (2015), O’Gorman (2017), and Baldin (2019).

10 Charlesworth and Chinkin (2000).

11 Charlesworth and Chinkin (2000).

12 See Otto 2015; Kapur (2019).

13 In that sense, Jones and Otto (2020).

14 See Grear (2015), 241.

15 Ito and Montini (2018).

16 On forms of oppression and dualism, see the work by the philosopher Plumwood (1993), 69.

17 Nixon (2011), 2.

18 “Upper- or middle-class, human, technologically and industrially ‘developed,’ male”: Gaard (1993), 1.

19 On the use of concepts in international law, Singh and d’Aspremont (Singh and d’Aspremonts 2019), 18.

20 Boyd (2017), xxxiv.

21 Plumwood (1993), 8.

22 See, among others, Warren (1990); Mies and Shiva (1993); Warren (1997); Mellor (1997); Mallory (2009); Bianchi (2012); Vakoch and Mickey (2018).

23 Collins (1974), 161.

24 Ruether (1975). Patterns of oppression have been perpetrated against indigenous peoples, on which this Reflection is not focusing. See, in particular, the work by Watson (2015) on aboriginal peoples, colonialism and international law.

25 Lear (1998). Carson published in the 1962 *Silent Spring*, where she highlighted the toxic connections between pesticides, environmental degradation, and inter-species health.

26 Adams (1991), 126.

27 Puleo (2017), 30.

28 Puleo (2017), 28.

29 Seager (1999), 164.

30 Malone (2015). From a domestic (Canadian) legal perspective, Hughes asked the reasons why “ecofeminism, both cultural and socialist, has developed critiques of third world development, pollution, reproductive technology, women’s health, hunting, animal experimentation, the nuclear industry, the medical and scientific establishments, and more [but not] by and large, directly engaged in a critique of environmental law:” Hughes (1995), 509.

31 See the multidisciplinary perspective invoked in the book edited by Warren (1997), which however misses the legal analysis. See also the landmark book Oppermann and Iovino (2017), which does not include a chapter on law.

32 On this aspect, see Kings (2017).

33 Natarajan and Khoday (2014).

34 Natarajan and Khoday (2014), 576.

35 Stone (2010), 23. His original article dates back to 1972. See also Nash (1989), who invoked the liberation of nature.

36 Taylor (1998), 40.

37 The definition is based on Taylor (1998), and Curry (2011).

38 See the pre-pandemic GA Res. 74/20 (Jan. 28) on global health, which only incidentally considered the environment. The WHO only recently acknowledged the connections between environment and global health (https://www.who.int/news-room/feature-stories/detail/who-manifesto-for-a-healthy-recovery-from-covid-19?fbclid=IwAR0uhZhJhWKe5X7MIsz50JuguORgiNtFTvJRCo5HhczEky4hGPSGPnC4kVY; accessed August 25, 2020). The very interesting proposals that have been elaborated so far rarely appreciate the centrality of the environment in the legal reasoning. See, among others, Taylor and Habibi (2020).

39 De Vido (forthcoming).

40 As argued by Faunce et al. (2014), 468: “issues of climate change, poverty, and lack of access to healthy food could then be viewed […] as global environmental pathologies to be remedied in part by commitment to environmental sustainability among systems of global health law.”

41 In the sense of connecting the human right to health and IHR, Toebes (2020).

42 Nollkaemper and Jacobs (2013). Scholtz (2013) identified the custodial element in cosmopolitan theory. See also Criddle (2019).

43 An interesting reflection on the role of states, based on Dworkin’s thought, in Besson (2020). She argued that we have “responsibility to preserve and improve the institutional order in which that *jus gentium* can flourish: the *mundus gentium*. It is that international institutional order we should tend to urgently, in order to make sure the world remains inhabitable by all the peoples who make it together” (136).

44 With regard to patterns of oppression in the Global South, see, for example, Agarwal (1992); Pandey (2013); Eaton and Lorentzen (2003).

45 Chinkin and Charlesworth (2000), 137.

46 Cançado Trindade (2010), 177.

47 Rodgers (2019), 714.

48 See, for example, Ecuadorian Constitutional Court Ruling, 017-12-SIN-CC, No. 0033-10-IN. See the analysis by Cano Pecharroman (2018), and the reflections on rivers’ personhood by Clark et al. (2018).

49 In this sense also the aforementioned advisory opinion of the Inter-American Court of Human Rights.

50 Huneeus (2020). See, using a political ontology perspective, Ruiz Serna (2017).

51 Huneeus (2020).

52 Huneeus (2020).

53 See, in that respect, Peters 2020, and bibliography therein.

54 Singer (1975), 243. See also Huffman (1992).

55 MacKinnon (2004), 271. As Peters (2016) interestingly reports, the plausibility of the comparison between sexism and specism emerges in the response to the 1792 manifesto, *A Vindication of the Rights of Women*, written by Mary Wollstonecraft. An English author wrote in response the pamphlet *A Vindication of the Rights of Brutes*, claiming that if one started down that path, one might as well grant rights to animals to emphasize how absurd it was to grant rights to women.

56 Boyd (2017), 57.

57 Boyd (2017), 57.

58 Peters (2016), 24.

59 Peters (2016), 25. The author has also encouraged “the legalization of some relevant rights for some non-human animals (notably the right to life, liberty, and freedom from torture), should be seriously considered—as a complement to the Universal Declaration of Human Rights” (Peters (2020), 118).

60 With regard to the responsibility to protect, see the interesting argument by Malone (2015), 1461.

61 SC Res No 2518/2020 (March 13, 2020).

62 ISIL has set oil wells on fire in Iraq, causing enormous environmental damages. UN official Erik Solheim declared that: “This ongoing ecocide is a recipe for a prolonged disaster. It makes living conditions dangerous and miserable, if not impossible. It will push countless people to join the unprecedented global refugee population. That’s why the environment needs to be placed at the centre of crisis response, conflict prevention and conflict resolution” (https://www.unenvironment.org/news-and-stories/press-release/mosul-battle-brings-environmental-damage-serious-impacts-health; accessed August 25, 2020).

63 Legality of the threat or use of nuclear weapons, Advisory Opinion, 1996 ICJ Report 226 (July 8). On the advisory opinion, see the comments, ex multis*,* by Bekker (1996), Ronzitti (1996), Grief (1997), and Matheson (1997).

64 Dissenting opinion of Judge Weeramantry, 433.
